# Microsatellite instability is highly prevalent in older patients with colorectal cancer

**DOI:** 10.3389/fsurg.2024.1288061

**Published:** 2024-03-27

**Authors:** Daniel Jakob, Valerie Orth, Daniel Gödde, Hubert Zirngibl, Peter C. Ambe

**Affiliations:** ^1^ Faculty of Medicine, Witten/Herdecke University, Witten, Germany; ^2^ Chair of Surgery II, Witten/Herdecke University, Witten, Germany; ^3^ Department of Pathology and Molecular Pathology, Witten/Herdecke University, Witten, Germany; ^4^ Department of General Surgery, Visceral surgery and Coloproctology, GFO Kliniken Rhein Berg, Vinzenz-Pallotti-Hospital Bensberg, Bergisch Gladbach, Germany

**Keywords:** early onset colorectal cancer, colorectal cancer, microsatellite instability (MSI), mismatch repair, immunohistochemistry

## Abstract

**Background:**

Clinical guidelines suggest screening of colorectal cancer (CRC) for microsatellite instability (MSI). However, microsatellite instability—high (MSI-H) CRC is not rare in older patients. This study aimed to investigate the prevalence of MSI-H CRC in an unselected population in an age-based manner.

**Material and methods:**

A retrospective analysis of data from patients undergoing radical surgery for CRC was performed. Only cases with results from MSI testing using immunochemistry (IHC) were analyzed. Age-based analyses were performed using two cut-off ages: 50 years. as stated in Amsterdam II guidelines, and 60 years. as outlined in the revised Bethesda criteria.

**Results:**

The study population included 343 (146 female and 197 male) patients with a median age of 70 years (range 21–90 years). The prevalence of MSI-H tumors in the entire cohort was 18.7%. The prevalence of MSI-H CRC was 22.5% in the group ≤50 years vs. 18.2% in the group >50 years using the age limit in the Amsterdam II guidelines. MSI-H CRC was present in 12.6% of the group aged ≤60 years compared to 20.6% in the control group >60 years.

**Conclusion:**

MSI screening of CRC based on age alone is associated with negative selection of a relevant number of cases. MSI-H CRC is also common in elderly patients, who may be negatively selected secondary to an age-based screening algorithm. Following the results of this study, screening based on clinical criteria should be omitted in favor of systematic screening as is already internationally practiced.

## Introduction

While the incidence of CRC seems to be decreasing in the general population over the last decades, the number of cases diagnosed at a young age has increased over the same period (1, 2). Generally, young age is defined by the Amsterdam II criteria as 50 years or younger (3, 4). Because the Amsterdam II criteria are very stringent, quite a number of MSI-H tumors went undetected. Thus the Amsterdam criteria were revised and the revised Bethesda criteria were defined (5). A central aspect of the revised clinical criteria was an increase of the age limit from 50 years to 60 years (5, 6). These clinical criteria were defined to identify individuals with high microsatellite instability (MSI-H) as a central feature of both hereditary nonpolyposis colorectal cancer (HNPCC) now known as “Lynch-like” syndrome and tumors within the spectrum of Lynch syndrome (7, 8). Tumors in both Lynch syndrome and Lynch-like syndrome develop secondary to mutations involving the Mismatch Repair (*MMR*) genes *MLH1*, *MSH2*, *MSH6*, and *PMS2* and possess a high degree of microsatellite instability (9–11). In both entities, CRC may develop at a very young age. The current German guidelines for the diagnosis and management of CRC recommend MSI testing in patients fulfilling the Amsterdam and Bethesda criteria (12). Thus, MSI testing is not routinely performed if these clinical characteristics are not met. More so, most clinicians tend to initiate MSI testing primarily when CRC is diagnosed at a young age (13, 14). Our clinical experience however indicates that MSI-H CRC may not be rare in elderly patients. We therefore intended to investigate the incidence of MSI-H CRC in an unselected population in an age-based manner.

## Methods

We performed a retrospective analysis of data from a prospectively maintained colorectal cancer database at a university hospital. The study received approval from the ethics commission at Witten/Herdecke University. The study population included all consecutive cases of CRC undergoing oncologic resection between January 2015 and December 2020. MSI screening on cancer specimens was performed using IHC for the gene products of the most relevant *MMR-genes*; *MLH1*, *MSH2*, *MSH6*, and *PMS2* as published by Shia et al. (15). Only patients with available IHC findings were included for analysis. Age-specific groups were created using the ages defined in the Amsterdam II (50 years) and Bethesda (60 years) criteria. The control group included all patients above the defined cut-off ages. Data analysis was performed using statistical package for social sciences (SPSS), IBM version 25. Continuous variables are reported using absolute numbers and percentages, while central tendencies are reported using medians and ranges. Analytic statistics were done using the chi-square test. All calculations were done with a 95% confidence interval and *p*-values <0.05 were reported as statistically significant.

## Results

**Figure 1:**
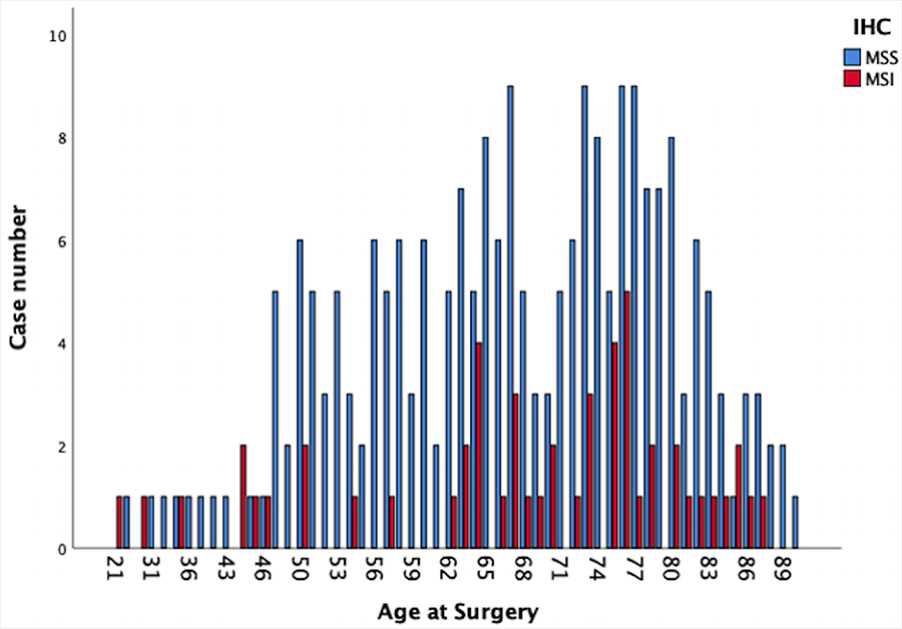
Distribution of the findings of IHC in the study collective.

**Figure 2:**
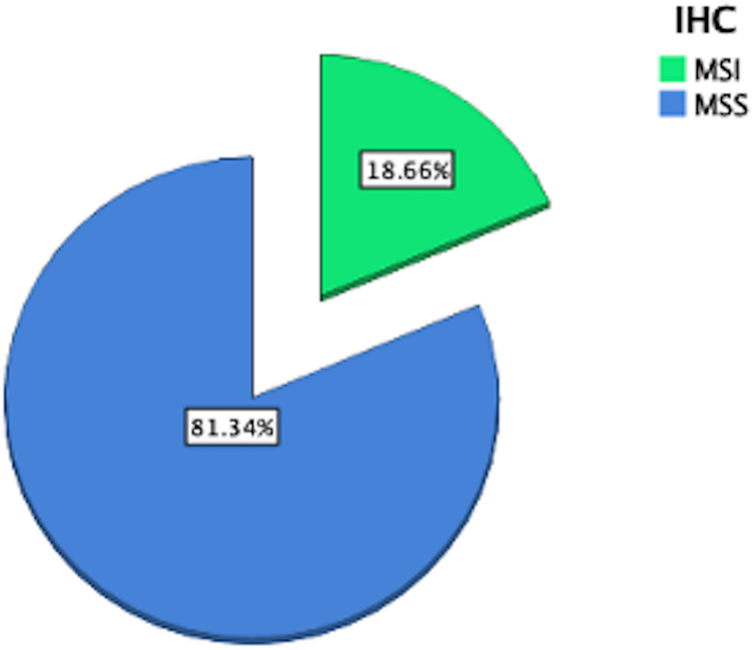
Prevalence of MSI-H tumors in the collective.

The study population included 343 (146 female and 197 male) patients with a median age of 70 years (range 21–90 years). The tumor was in the right colon in 141 cases (41.1%), while the left colon including the rectum was involved in 202 cases (58.9%). Figure 1 demonstrates the distribution of IHC results for MSI in the study population, while the prevalence of MSI-H tumors is reported in Figure 2.

**Figure 3:**
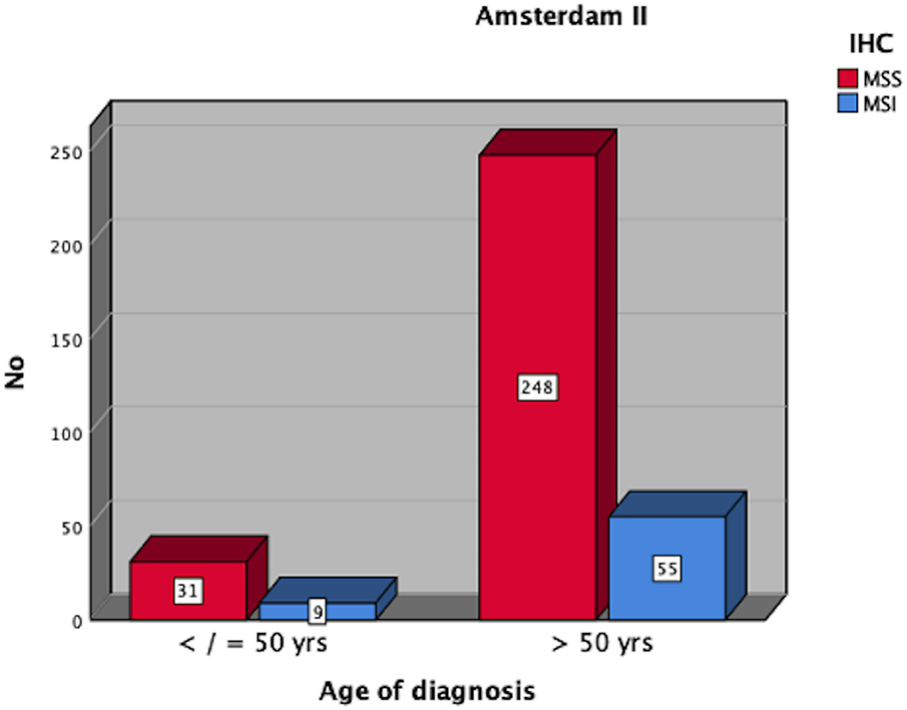
Distribution of MSI-H CRC amongst patients ≤50 vs. >50 years.

**Figure 4:**
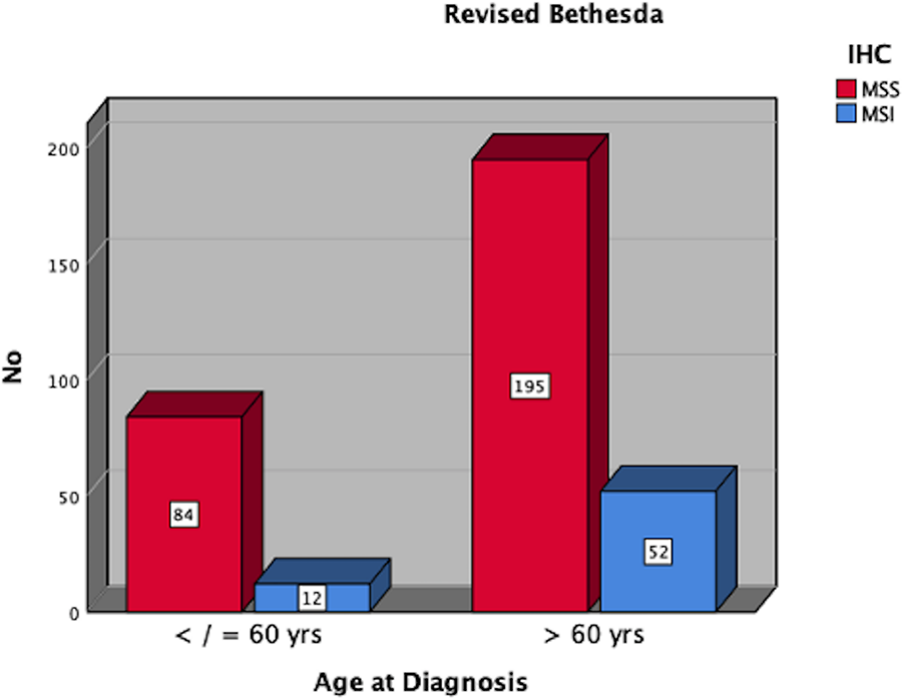
Distribution of MSI-H tumors in patients ≤60 vs. >60 years.

Table 1 represents the results of the first age-based analysis using the 50 years as cut-off as defined in the Amsterdam criteria, while the prevalence of MSI-H tumors for this is presented in Figure 3. The clinicopathological findings from the second analysis with 60 years as cut-off, based on the revised Bethesda criteria are presented in Table 2, while the corresponding prevalence of MSI-H tumors is demonstrated in Figure 4.

**Table 1:** Age-based analysis with cut-off at 50 years.

Characteristics	Age ≤ 50 years	Age > 50 years
	*N* = 40	*N* = 303
Sex		
Female	19 (47.5%)	127 (41.9%)
Male	21 (52.5%)	176 (58.1%)
Age		
Median	48 years	72 years
Range	21–50 years	51–90 years
Location of CRC		
Right colon	14 (35.0%)	127 (41.9%)
Left colon	26 (65.0%)	176 (58.1%)
UICC stages		
I–II	185 (61.1%)	38 (54.2%)
III	75 (24.8%)	22 (31.4%)
IV	43 (14.2%)	10 (14.3%)
MSI-status		
MSS	31 (77.5%)	248 (81.8%)
MSI	9 (22.5%)	55 (18.2%)

**Table 2:** Clinicopathological features based on age 60 years.

Characteristics	Age ≤ 60 years	Age > 60 years	*p*-Value
Sex	*N* = 96	*N* = 247	
Female	42 (43.7%)	104 (42.1%)	>0.05
Male	54 (56.3%)	143 (57.9%)	
Age			
Median	52 years	74 years	0.03
Range	21–60 years	61–90 years	
UICC Stages			
I–II	58 (61.7%)	152 (61.8%)	>0.05
III	23 (22.3%)	56 (22.8%)	
IV	15 (16.0%)	39 (15.4%)	
MSI-status			
MSS	84 (87.4%)	196 (79.4%)	>0.05
MSI	12 (12.6%)	51 (20.6%)	

## Discussion

Early onset CRC is defined in Amsterdam criteria as CRC before the age of 50 years and may be secondary to hereditary cancer predisposition. Another age limit (60 years) commonly used in the literature is defined revised Bethesda criteria. Both clinical criteria aimed at selecting individuals for microsatellite instability screening in the setting of CRC. However, MSI-H CRC is not a rare finding in older individuals (>60 years) with CRC. This study investigated the prevalence of MSI-H CRC in patients regarding the age at surgery using the two cut-off ages (50 and 60 years). The prevalence of MSI-H CRC in this study was 18.7%. The prevalence of MSI-H CRC in patients ≤50 years as defined in the Amsterdam II criteria was 22.5%, while MSI-H CRC was found in 12.6% of patients ≤60 years.

The prevalence of MSI-H CRC in this study was slightly higher in patients ≤50 years compared to controls (>50 years) 22.5% vs. 18.2%. This was not statistically significant. This finding must be interpreted with caution since numerically more cases of MSI-H CRC were found in the group >50 years compared to the younger cohort (9/40 vs. 55/303). A total of 55 cases of MSI-H CRC would have been undetected in this population if the Amsterdam II criteria alone had been used as the sole prerequisite for MSI testing.

Increasing the cut-off age to 60 years as spelled out in the revised Bethesda criteria was associated with a drop in the prevalence of MSI-H tumors from 22.5% in patients ≤50 years to 12.6% in the group ≤60 years The huge drop in the prevalence of MSI-H CRC amongst the two age groups is a simple effect of the number of cases associated with the age dynamic. Increasing the age from 50 to 60 years led to a marked increase in the size of the population from 40 to 96 cases. Although statistically not significant, the prevalence of MSI-H CRC was higher in the older group >60 years (20.6%) in comparison to the age group under 60 years (12.6%). This finding opposes the recommendation by Chou et al. to perform MSI screening for right-sided CRC in individuals ≤60 years not only regarding age but also with regard to the cancer location (16).

The findings of this study indicate that initiation of MSI screening in CRC based on clinically defined criteria bears a great risk of missing quite a large portion of potential cases with MSI-H CRC. Our findings are in line with the current literature regarding the poor performance of both the Amsterdam and the revised Bethesda criteria as selection tools for MSI screening (3, 4, 6). These findings should be seen as an argumentation to leave the dogma of fulfillment of clinical criteria and move on to systematic screening of colorectal neoplasia for MSI, especially since these clinical criteria were aimed at identifying individuals with possible germline mutations at risk for Lynch syndrome (17–19).

The results of this study indicate that the common practice of “red flag raising” and initiation of MSI-testing preferably in younger individuals with CRC leads to a high probability of undiagnosed cases with MSI-H CRC. This finding is in accordance with the data published by Poynter et al. indicating that advanced age is a strong predictor for MSI-H CRC secondary to MLH1 methylation (20). More so, omitting MSI screening due to failure to fulfill clinical criteria may be detrimental to patients with stage II and III CRC regarding the need and choice of additive chemotherapy, especially regarding responsiveness to 5-FU-based regimes (21, 22). This may be a possible explanation for the previously reported poor outcome of CRC in young patients with advanced CRC (23).

A major limitation of this study is the retrospective design. Many cases needed to be excluded due to missing data. This led to a reduction in the study population. MSI in this study was investigated using immunohistochemistry only. Although there is a high concordance between PCR testing and IHC, there is still a possibility that some cases of MSI-H went undiagnosed via IHC alone. It is therefore fair to question whether the results recorded in this study may be reproducible in a larger population. More so, findings from genetic counselling and testing for possible germline mutations to diagnose or exclude Lynch syndrome in MSI-H cases were not generally performed and therefore could not be reported.

## Conclusion

MSI screening of CRC based on age alone is associated with negative selection of a relevant number of cases. MSI-H CRC is also common in elderly patients, who may be negatively selected secondary to an age-based screening algorithm. Following the results of this study, screening based on clinical criteria should be omitted in favor of systematic screening as is already internationally practiced.

## Data Availability

The raw data supporting the conclusions of this article will be made available by the authors, without undue reservation.
